# Child temperamental reactivity and self-regulation effects on attentional biases

**DOI:** 10.3389/fpsyg.2014.00922

**Published:** 2014-08-25

**Authors:** Georgiana Susa, Oana Benga, Irina Pitica, Mircea Miclea

**Affiliations:** ^1^Developmental Psychology Lab, Department of Psychology, Babes-Bolyai UniversityCluj-Napoca, Romania; ^2^Department of Psychology, Babes-Bolyai UniversityCluj-Napoca, Romania

**Keywords:** child temperament, fear, attentional control, attentional biases, anxiety

## Abstract

This study examined the effects of individual differences in temperamental reactivity (fear) and self-regulation (attentional control) on attentional biases toward threat in a sample of school-aged children (age range was between 9 years 1 month and 13 years 10 months). Attentional biases were assessed with pictorial Dot-probe task, comparing attention allocation toward angry (threat-related) vs. neutral and happy faces. Children also completed self-report temperamental measures of fear and attentional control. We compared attentional bias scores in 4 groups of children: high/low fear and high/low attentional control. Results indicated that, in the case of children with high fear and low attentional control, attention was significantly biased toward angry faces compared with children who had low fear and low attentional control. Findings are discussed in terms of the moderating role of individual differences in attentional control in the context of threat, anxiety-related attentional biases in children.

## Introduction

Cognitive theories have proposed that anxious individuals tend to direct their attention toward threatening information during early stages of processing (Beck and Clark, 1997). Specific theoretical accounts of attentional biases toward threat state that biases could appear as a result of exaggerated pre-attentional threat evaluation (Williams et al., 1988; Mogg and Bradley, 1998), but also as a result of a failure of effortful strategies to focus on task-related rather than threat stimuli (Mathews and MacKintosh, 1998; Cisler and Koster, 2010).

A lot of research in this field has been conducted with adults and indicates that the tendency to attend toward threatening information is associated with both anxiety disorders and non-clinical high levels of anxiety (Bar-Haim et al., 2007). Childhood investigations have also examined the association between attentional biases toward threat and anxiety, with different experimental paradigms, from reaction time (e.g., Dot-probe and emotional Stroop tasks) to eye-tracking (see In-Albon et al., 2010), but results are less straightforward.

Attentional biases were found in clinical child populations, when using verbal (neutral vs. threat words) or non-verbal stimuli (negative, e.g., angry or fearful vs. neutral and positive facial expressions) (e.g., Vasey et al., 1995; Dalgleish et al., 2003; Brotman et al., 2007; Roy et al., 2008; Waters et al., 2010a). Moreover, in non-clinical samples, some studies comparing children categorized as highly anxious vs. non-anxious reported attentional biases characteristic of the first category, when using both the emotional Stroop task (Martin and Jones, 1995; Richards et al., 2000) and the Dot-probe task (Vasey et al., 1996). A recent study compared low-anxious and moderately-anxious children aged 7–14 years in an emotional Stroop task as well as in a face Dot-probe task. Authors reported enhanced processing bias for angry faces compared to neutral ones on the Stroop task as being characteristic of younger moderately anxious children, mean age 9 (Reinholdt-Dunne et al., 2012). The same children showed enhanced negative as well as positive processing biases in the Dot-probe task. However, older moderately anxious children (mean age 11) had lessened anxiety-related threat bias, result interpreted as a consequence of heightened abilities of executive control which are growing with age.

In spite of the above-mentioned evidence for threat-related attentional biases in anxious children, several studies reported challenging results. For example, whereas studies generally found evidence for attentional biases toward threat (e.g., Roy et al., 2008), some authors reported a pattern of attentional biases away from threatening stimuli (e.g., Monk et al., 2006). Furthermore, some studies revealed that attentional biases are present in both anxious and non-anxious children (e.g., Eschenbeck et al., 2004), whereas others found attentional biases toward threat only in children who were clinically diagnosed with anxiety disorders (Roy et al., 2008). Also, results with regard to attentional biases for positive stimuli (happy faces) are mixed and rather ambiguous (e.g., Waters et al., 2008, 2010b; Reinholdt-Dunne et al., 2012).

In conclusion, there seem to be diverging, thus provocative findings on threat-related attentional biases in children. In addition, an essential question addresses the role of these attentional biases on the onset and maintenance of clinical anxiety. Both lines of inquiry recently encouraged the growth of developmental perspectives that look at potential routes through which such biases toward threat might emerge in children and further sustain the development of psychopathology.

### Temperament and attentional biases

A fundamental theoretical position suggests that temperamental factors might predispose children to manifest attentional biases toward threat (Lonigan et al., 2004; Helzer et al., 2009; Pine et al., 2009).

According to the model of Lonigan et al. (2004), temperamental traits involving sensitivity toward threat (e.g., negative affectivity, behavioral inhibition) are considered critical in making children prone to allocate their attention toward threatening information. Negative affectivity represents a reactive dimension of temperament, within the theoretical framework of Rothbart (Rothbart and Derryberry, 1981), and is defined as a tendency to experience negative emotions, such as fear, sadness, and anger. This reactive dimension of temperament describes the individual's responsiveness to environmental stimuli, in terms of the extent to which negative affect and avoidance behaviors vs. positive affect and approach behaviors are elicited (Henderson and Wachs, 2007; Posner and Rothbart, 2007). Also, this temperamental dimension is characterized by sensitivity to negative stimuli, physiological arousal, and emotional distress (Ingram and Price, 2010). A different approach, first advanced by Kagan (Kagan et al., 1987; Schmidt et al., 1997; Fox et al., 2005) delineates the temperamental profile of behavior inhibition, characterized by fear of novelty, social reticence, and further proneness to internalizing disorders, thus partly overlapping with a high negative affectivity temperamental profile.

Along with such temperamental traits that might underlie an individual's sensitivity to threat, the model of Lonigan et al. (2004) takes into consideration the self-regulatory system, serving to modulate reactivity, which is described in Rothbart's theory by the dimension of effortful control. This regulative dimension of temperament includes processes such as inhibition, avoidance, and attentional self-regulation (Rothbart and Bates, 2006; Posner and Rothbart, 2007).

A specific component of the self-regulatory temperamental dimension of effortful control is that of attentional control. Temperamental attentional control reflects stable individual differences in the ability to focus and shift attention with ease vs. harder or less natural (Posner and Rothbart, 2000; Simonds et al., 2007), assumed to have its cognitive underpinnings in the executive or anterior attention system (Posner and DiGirolamo, 1998; further developments in Fan et al., 2002; Rueda et al., 2005); but see also a recent approach advanced by Zhou et al. (2012) that considers temperamental attentional control similar to executive control. A critical function of attentional control is to disengage attention from threatening irrelevant information and to keep attention focused on task relevant stimuli. Thus, attentional control is considered especially important in emotion regulation in general and in reducing internalizing symptoms such as anxiety and sadness in particular (Eisenberg et al., 2000; Calkins and Fox, 2002; Bell and Calkins, 2012).

The model advanced by Lonigan and collaborators suggests that attentional biases for threat can be seen in children with high levels of negative affectivity who also have low effortful control and more specifically low attentional control. From a developmental perspective, attentional biases are expected to emerge early in life in children born with an underlying anxiety predisposition, such as high levels of negative affectivity, coupled with low levels of effortful control. Moreover, such biases are expected to further play a mediating role in the relation between temperament and the development of anxiety disorders. Although this theoretical model is compelling, to date there are few studies that specifically examined the link between temperament and attentional biases toward threat. Initial results are nevertheless promising, suggesting that children with fearful temperament—an important aspect of negative affectivity—tend to preferentially allocate their attention toward threat (White et al., 2010). Moreover, children with high levels of both negative affectivity and attentional control do not show attention biases toward threatening words, while children with high levels of negative affectivity coupled with low attentional control present vigilance toward these stimuli, as demonstrated by Lonigan and Vasey (2009) with a Dot-probe task using neutral and threatening words. Efficient attentional control processes may help children with fearful temperament inhibit the processing of task-irrelevant information and focus on the task-relevant information in the environment (see Pine et al., 2009 for a theoretical interpretation of data). High attentional control can thus enable individuals to override initial reactive attentional biases, and further serve as a protective factor against the development of anxiety disorders, as demonstrated by Vervoort et al. (2011) in a study with adolescents. In addition, empirical research on anxiety and attention, in adults with high levels of trait anxiety, has provided evidence that impaired attentional control might underlie attentional biases. Individuals with high trait anxiety but low levels of self-reported attentional control maintained a vigilance bias toward threat cues even at 500 ms, whereas those with high levels of attentional control shifted attention away from the threat location (Derryberry and Reed, 2002). From a developmental perspective, the longitudinal study conducted by Hardee et al. (2013) showed, in an event-related functional magnetic resonance imaging with Dot-probe task, that young adults characterized in early childhood with behavioral inhibition (BI) exhibited greater strength in threat-related connectivity than non-behaviorally inhibited young adults. Specifically, young adults with a history of BI manifested greater negative connectivity between amygdala and two frontal regions (dorsolateral prefrontal cortex and anterior insula) during trials containing angry faces compared to neutral faces. Also, amygdala—insula connectivity interacted with childhood BI to predict young adult internalizing symptoms.

All these results highlight the importance of analyzing the role of regulative temperamental factors, such as attentional control processes, in junction with the role of reactive temperamental traits, like negative affectivity or fear. Also, these results converge with cognitive theoretical accounts of attentional biases (Mogg and Bradley, 1998; Cisler and Koster, 2010), in suggesting that exaggerated engagement of attention to threat is, on the one hand, linked with an automatic/ pre-attentional threat detection mechanism, which is extremely sensitive in people born with an underlying anxiety predisposition, and on the other hand, with a failure of effortful strategies such as temperamental attentional control to regulate these initial automatic tendencies. However, in children, the relation between temperamental variables (both reactive and regulative) and attention toward threat has been under-investigated (with the exception of the studies done by Helzer et al., 2009; Lonigan and Vasey, 2009). Moreover, childhood research discussed above used threat-related words rather than emotion-eliciting pictorial stimuli. But pictorial stimuli, for example emotional facial expressions, are considered more ecological, compared to words which are limited in threat value and more open to subjective interpretability (Mogg and Bradley, 1999).

### Present study

In the present study, we aimed to examine the effects of individual differences in temperamental fear and attentional control processes on attention allocation toward threat, in children aged 9–14. Based on the assumed strengths of the Lonigan model, of greatest interest was the interaction effect between temperamental fear and temperamental attentional control on attentional allocation toward threatening information. We examined attentional biases toward threatening facial expressions, in order to fill the gap in existing research relative to attentional processing of more ecological stimuli.

Our hypotheses were the following: first, regarding the influence of temperamental fear, we expected that children with higher levels of fear would show enhanced attentional allocation toward angry faces, compared to children with low fear; second, we expected that temperamental attentional control might moderate the relation between temperamental fear and threat-related attentional biases. Specifically, only fearful children with low attentional control were expected to significantly bias their attention toward angry faces.

We believe that, from a theoretical perspective, the present study will extend the existing research on the relation between temperamental predispositions and threat related attentional biases, by adding information about the mechanisms underlying the emergence of attentional biases. Moreover, such an approach may help to inform prevention strategies regarding children who are prone to develop anxiety disorders. Such strategies could be designed to increase their resilience by means of attentional control training procedures.

## Materials and methods

### Participants

Our sample initially consisted of 163 school-aged Romanian children. This sample was part of a larger screening study conducted in our laboratory, concerning the relations between attentional biases and anxiety symptoms in children and adolescents. We obtained parental written informed consent and verbal consent from each child before the testing. In the current study, we included only children for whom we had both reaction time data and self-report data for temperamental fear, temperamental attentional control, and non-clinical anxiety symptoms. Consequently, 5 children were excluded from this study due to scheduling difficulties that lead to missing data on the measure of anxiety symptoms. The final sample included 158 participants, 70 of them girls. The age range of these participants was between 9 years 1 month and 13 years 10 months. Mean age of this sample was 11 years and 3 months. All children included in the sample were free of any clinical psychological diagnosis, as reported by teachers and school psychologists. Also their vision was normal or corrected.

### Materials

The questionnaires employed in this study to assess temperamental variables were the fear subscale from the Early Adolescent Temperament Questionnaire-Revised (EATQ-R; Ellis and Rothbart, 2001) and the child version of the Attentional Control Scale (ACS-C; Derryberry and Reed, 2002). Even though EATQ-R assesses various components of temperament, we selected only the fear subscale, since our research question is grounded on previous data showing that children with temperamental fear– an important aspect of negative affectivity—tend to preferentially allocate their attention toward threat (White et al., 2010). EATQ-R also contains an attentional control subscale, but this has only 8 items compared to ACS-C that has 20, thus being a more comprehensive measure of this temperamental dimension. The rationale for choosing these two particular temperamental scales was that both were developed based on Rothbart's model of temperament, which represents the conceptual temperamental framework of the Lonigan model.

The EATQ-R is a measure of temperament designed to be used with 9 to 15 year old children and adolescents. We selected the fear subscale of this questionnaire to assess self-reported temperamental fear in children. The fear subscale reflects the tendency to experience unpleasant anticipation of distress (Helzer et al., 2009). Children are asked to rate each item on a 5-point Likert scale and assess the frequency with which the item is true or false in their case. Some examples of items from the fear subscale of the EATQ-R are: “I worry about getting into trouble” or “I worry about my parent(s) dying or leaving me.” The EATQ-R was adapted for use with Romanian children through the following steps: (a) the scale was translated from English into Romanian by an expert in the field of temperament and development; (b) in order to verify that the original conceptual content has been preserved in the Romanian version, the Romanian translation was back translated to English by a different expert with proficiency level English as a foreign language qualifications; (c) the Romanian translation of the EATQ-R was employed in a pilot study with children aged between 9 and 14, to check that the language used was accessible to this age group.

In the present study we used only the fear subscale of EATQ-R that showed moderate internal consistency, Cronbach's Alpha being 0.69 in our sample of children.

The ACS-C is a self-report 20-item scale that evaluates children's ability to focus and shift attention. The scale contains 10 items that measure the ability to focus attention (e.g., “When I concentrate myself, I do not notice what is happening in the room around me”) and another 10 items that measure the ability to shift attention (e.g., “When I am doing something, I can easily stop and switch to some other task”). Children are answering the items by reporting how frequently certain things happen to them and they respond on a 4-point Likert scale. A good capacity of attentional control is reflected by higher scores obtained on this scale. Different studies conducted with different samples report good internal consistency of the ACS-C (Muris et al., 2004, 2007). The ACS-C was adapted for use with Romanian children through the same procedure described in the case of the EATQ-R adaptation. In the present study the ACS-C showed good internal consistency as Cronbach's Alpha coefficient reached 0.80.

The Spence Child Anxiety Scale was used to measure anxiety symptoms (SCAS; Spence, 1998). The SCAS child version is a 38-item self-report anxiety measure. This questionnaire asks children to rate how frequently they experience the situations described by each item using a 4-point Likert scale: 1- Never, 2- Sometimes, 3- Often, and 4- Always. By summing the scores from all items a total score can be computed. Also the SCAS offers subscale scores based on the anxiety disorder categories indexed in the Diagnostic and Statistical Manual for Mental Disorders IV (American Psychiatric Association, 1994). The subscales assess social anxiety, separation anxiety, obsessive-compulsive disorder, panic and agoraphobia, physical injury fears, and generalized anxiety. The Romanian version of the SCAS has been adapted for use with Romanian children through the same procedure described in the case of the EATQ-R adaptation and is currently under validation (Benga et al., in press). Studies conducted with other samples reported good psychometric properties (Spence, 1998; Spence et al., 2003). In the current study we obtained good internal consistency for the global scale. Cronbach's Alpha coefficient reached 0.85.

Attentional biases were measured with a pictorial version of the Dot-probe task, adapted from Bradley et al. (1998) and Susa et al. (2012). During the task, the children were seeing a series of trials on the computer screen. Each trial consisted of the following events: the fixation point in the center of the screen for 500 ms, a pair of pictures showing human facial expressions for 500 ms, the probe (a star) replacing one of the pictures, and a blank screen as a pause for 500 ms. The probe was displayed on the screen until a response was given. The facial stimuli were 64 images selected from a pool of 96 images from the following sets: 22 from the NimStim (Tottenham et al., 2009; http://www.macbrain.org/resources.htm)^1^, 5 from the Ekman stimuli set (Ekman and Friesen, 1976) and 37 from the stimuli developed by Mogg and Bradley (Bradley et al., 1998). We combined stimuli from different sets in order to present only Caucasian persons, since Romanian children are mostly familiar with this race. In the current study, we did not ask children to complete an emotion recognition task due to time constraints. However, we recruited a second group of children having, the same age as participants from our initial sample, and we tested whether they can accurately identify the emotional meaning (i.e., recognition accuracy) and rate the emotional intensity of the facial stimuli used within the Dot-Probe task (the description of this study and its results can be found in the Supplemental Material)^2^. To our knowledge, there are no other published validation studies with children for these picture sets. Though, face stimuli from the three databases were in general used by all previous studies conducted with children (e.g., for Ekman stimuli set see Szpunar and Young, 2012; for Mogg and Bradley stimuli see Roy et al., 2008; for NimStim see Tottenham et al., 2011) and data seem to support the view that children can recognize the emotional meaning at adult-like levels. Two types of threatening facial expressions were used in previous studies, in order to assess attentional allocation toward emotional facial expressions, namely fearful and angry faces. To our knowledge, there are no studies reported in the literature that compared attentional biases to fearful and angry faces in anxious children. However, a study conducted by Mogg et al. (2007) with an adult sample showed that fearful and angry faces elicited similar attentional biases in high-anxious individuals. In general, fearful faces were used by neuroimaging research (e.g., Whalen et al., 2001) since they seem to elicit more amygdala activity, given that they are more ambiguous (e.g., they signify the presence of danger, but do not provide information about its source). In contrast, angry faces were predominantly employed by Dot-probe studies, in which research questions were framed in terms of cognitive models of anxiety and which investigated the influence of anxiety on attentional allocation toward threat. Therefore, to facilitate the comparison and interpretation of our data with previous Dot-probe studies, we chose to present angry faces in order to assess attentional biases for threat.

### Procedure

Data from both the questionnaires and the Dot-probe task were collected from children in two schools, in the presence of a research assistant. Children who voluntarily consented to participate were asked to have their parents sign the informed consent form. In order to prevent children's fatigue, questionnaire data were collected first and then, approximately 2 weeks after, children completed the Dot-probe task.

For the Dot-probe task, children were seated in front of the computer at a distance of approximately 40 cm from the screen. At this distance, they were able to comfortably reach the laptop keyboard throughout the task. The task was presented to the children as a computer game and they were asked to read the instructions displayed. Participants were instructed to press key A when the probe replaced the picture on the left side of the screen and key L when the probe replaced the picture on the right side of the screen (on a QWERTY keyboard). Before starting the task, the research assistant summarized for each child what he or she was asked to do. For each child, the program presented the picture pairs in random order. At the end, each child received positive feedback and a small reward.

During the Dot-probe task all children included in the analysis completed 160 experimental trials and 8 practice trials. There were a total of 80 pairs of stimuli, 32 of them showing angry-neutral facial expressions, 32 showing happy-neutral facial expressions and 16 pairs showing neutral- neutral facial expressions. By including neutral-neutral pairs, we could analyze the two mechanisms of attentional biases discussed in the literature: attentional faster engagement by angry faces or difficulty of disengagement from angry faces (e.g., Koster et al., 2004). Also, in this way we could control that our reaction time data are not better explained by behavioral interference effects (Wolters et al., 2012).

## Results

### Preliminary analyses

#### Dot-probe data preparation

Reaction time data for each participant were screened and trials with response time less than 200 ms or greater than 1500 ms were eliminated from further analyses (a total of 0.67% of the total data). Trials with reaction times greater than 1500 ms are considered to represent outliers and are likely attributable to error, therefore not excluding these reaction times would have influenced individually trimmed means (Oehlberg et al., 2012). It is highly probable that during such trials children were not paying close attention to the displayed stimuli. Mean accuracy level for the whole sample was 98.73% of all responses. Trials with incorrect responses were excluded from the reaction time analysis.

We then computed an attentional bias score for each child. These bias scores were calculated by subtracting mean reaction times for congruent trials from mean reaction times for incongruent trials (Mogg and Bradley, 1999). The difference between congruent and incongruent trials is the location of the probe relative to the emotional face. In congruent trials, the probe appeared on the same location as the emotional face (angry or happy), and for incongruent trials the probe appeared on the same location as the neutral face. Positive values indicate a vigilance bias and negative values indicate an avoidance bias for emotional faces. The same analysis was carried out both for angry as well as for happy expression trials.

#### Questionnaire total sample and group characteristics

The total mean fear score on the fear subscale of the EATQ, for the whole sample of children in this study was 2.85 (*SD* = 0.75; minimum score 1, maximum score 4.67). This is similar to the mean reported by Muris and Meesters (2009) in a community sample of Belgian and Dutch children aged 8 to 14 (*M* = 2.69, *SD* = 0.77).

The total mean attentional control score on the ACS-C for the whole sample of children in this study was 26.74 (*SD* = 6.14; minimum score 11, maximum score 44). This is somewhat different from the mean reported by Muris et al. (2004) in a community sample of Dutch children aged 8 to 13 (*M* = 34, *SD* = 8.1).

Mean anxiety score on the SCAS, in the whole sample, was 29.28 (*SD* = 15.88; minimum score 1, maximum score 81). The Romanian version of the SCAS is currently under validation but preliminary data (Benga et al., in press) from a sample of 300 children aged between 9 and 15 years showed a similar SCAS mean score (*M* = 29.60, *SD* = 15.43; minimum score 1, maximum score 82).

By using the median split of the ratings of children's fear level (median value was 3) and attentional control level (median value was 20) we formed four groups (see Table 1 for descriptive data within each condition): a high fear, high attentional control group (HFHAC); a high fear, low attentional control group (HFLAC); a low fear, high attentional control group (LFHAC); and a low fear, low attentional control group (LFLAC). The four groups did not significantly differ in age, *F*_(3, 154)_ = 0.87, *ns* or anxiety scores, *F*_(3, 154)_ = 1.10, *ns*. Also, the four groups did not significantly differ in overall reaction times in the Dot-probe task, *F*_(3, 154)_ = 0.49, *ns*, or in accuracy, *F*_(3, 154)_ = 0.27, *ns*.

**Table 1:** **Descriptive data for each group as a function of both temperamental dimension (fear and attentional control), gender and age**.

**Group**	***N***	**Gender**		**Age**			**Fear**		**Attentional control**		**Age in months**	
		**Girls**	**Boys**	**9–10**	**11–12**	**13–14**	**Mean**	***SD***	**Mean**	***SD***	**Mean**	***SD***
HFHAC^a^	23	11	12	9	9	5	3.55	0.29	23.39	2.13	133.39	15.20
		47.8%	52.2%	39.13%	39.13%	21.74%						
HFLAC^b^	43	21	22	11	25	7	3.63	0.39	15.60	2.92	136.30	12.69
		48.84%	51.16%	25.58%	58.14%	16.28%						
LFHAC^c^	53	21	32	15	21	17	2.35	0.54	24.28	2.09	137.98	15.40
		39.6%	60.4%	28.31%	39.62%	32.07%						
LFLAC^d^	39	17	22	11	19	9	2.39	0.49	16.92	3.62	136.38	14.65
		43.6%	56.4%	28.20%	48.72%	23.08%						

a *High Fear, High Attentional Control*.

b *High Fear, Low Attentional Control*.

c *Low Fear, High Attentional Control*.

d *Low Fear, Low Attentional Control*.

In order to control for behavioral interference effects in the Dot-probe task, we conducted a preliminary 2 × 2 × 2 ANCOVA (Fear x Attentional control × Face valence) analysis with Age as a covariate, for comparing reaction times in the four groups between all conditions, with neutral faces collapsed and all conditions with angry faces collapsed. We ran separately a 2 × 2 × 2 ANCOVA analysis between all conditions with neutral faces and all conditions with happy faces. Results indicated no significant differences in overall reaction times when face stimuli were neutral vs. angry, *F*_(5, 152)_ = 0.23, *ns*, or when face stimuli were neutral vs. happy, *F*_(5, 152)_ = 1.99, *ns*.

### Main analyses

The theoretical focus of our study was on estimating the impact of temperamental variables and of their interaction on attentional biases, while controlling for the effect of other variables that may influence both the measured independent and the dependent variables. Therefore, because anxiety may influence attention to threat and individual differences in temperamental traits are associated with anxiety, we included anxiety as a covariate. Also, the quasi-experimental design of the present study, in which participants were not randomly assigned to groups, requires the inclusion of this covariate (Yzerbyt et al., 2004). Besides anxiety, we also included age as a covariate in the design, since our sample covered quite a wide age range and this is a factor known to influence reaction times (Anderson et al., 1997; Iida et al., 2010).

#### Analysis of covariance

As our hypotheses were concerned with differences between groups, we conducted a mixed ANCOVA with Emotion valence (angry or happy) as a within-subjects factor, Fear and Attentional Control levels as between-subjects factors, and Age (in months) and Anxiety as covariates. This analysis indicated no significant main effect, but a significant three-way interaction effect of Emotion valence by Fear level by Attentional Control level, *F*_(5, 151)_ = 7.72, *p* < 0.01, partial η^2^ = 0.05 (Bonferroni correction applied). In order to understand the three-way interaction we completed two separate ANCOVAs, one for the angry bias scores and another for the happy bias scores.

The 2 × 2 ANCOVA (Age and Anxiety as covariates) for angry bias scores indicated a significant interaction effect of Fear and Attentional Control levels on bias scores, *F*_(3, 152)_ = 5.58, *p* = 0.01, partial η^2^ = 0.03 (Bonferroni correction applied). No main effects of Fear, *F*_(3, 152)_ = 1.22, *ns*, Attentional Control, *F*_(3, 152)_ = 0.05, *ns*, Age, *F*_(3, 152)_ = 0.002, *ns*, or Anxiety, *F*_(3, 152)_ = 0.20, *ns*, reached significance^3^. As such, highly fearful children who also have high levels of attentional control seem to have weaker attentional biases toward threat, as compared to highly fearful children with low levels of attentional control (see Table 2 for means and standard deviations).

**Table 2:** **Mean threat reaction times for each condition and mean bias scores for the four groups (with standard deviations in parentheses)**.

**Type of emotion**	**Reaction times**		**Attentional bias score**
	**Emotional congruent**	**Emotional incongruent**	
**HIGH FEAR, HIGH ATTENTIONAL CONTROL GROUP**			
Angry	470.98 (69.92)	467.34 (63.97)	−3.64 (29.83)
Happy	463.02 (62.36)	467.76 (60.88)	4.74 (26.74)
**HIGH FEAR, LOW ATTENTIONAL CONTROL GROUP**			
Angry	489.30 (59.56)	497.83 (61.78)	8.53 (27.14)
Happy	491.91 (60.94)	490.43 (56.28)	−1.48 (24.52)
**LOW FEAR, HIGH ATTENTIONAL CONTROL GROUP**			
Angry	479.83 (72.47)	480.17 (77.82)	0.34 (25.24)
Happy	475.29 (73.24)	470.95 (74.75)	−4.34 (28.65)
**LOW FEAR, LOW ATTENTIONAL CONTROL GROUP**			
Angry	491.19 (83.29)	482.42 (85.14)	−8.77 (24.19)
Happy	486.62 (85.17)	489.37 (84.85)	2.75 (23.63)

Further we decomposed the interaction effect with follow-up *t*-tests. We looked at the main effect of fear on attentional biases toward angry faces as a function of attentional control. When comparing the low fear low attentional control group with the high fear, low attentional control group, for threat bias scores we observed a significant difference, *t*_(80)_ = −3.03, *p* = 0.003, *d* = 0.68 two-tailed. Inspecting the means from Table 2, we can see that children with high temperamental fear and low attentional control were significantly vigilant toward angry faces. When we looked at the other two groups and compared children with low fear and high attentional control to children with high fear and high attentional control, we observed a non-significant effect, *t*_(74)_ = 0.59, *ns*.

**Figure 1:**
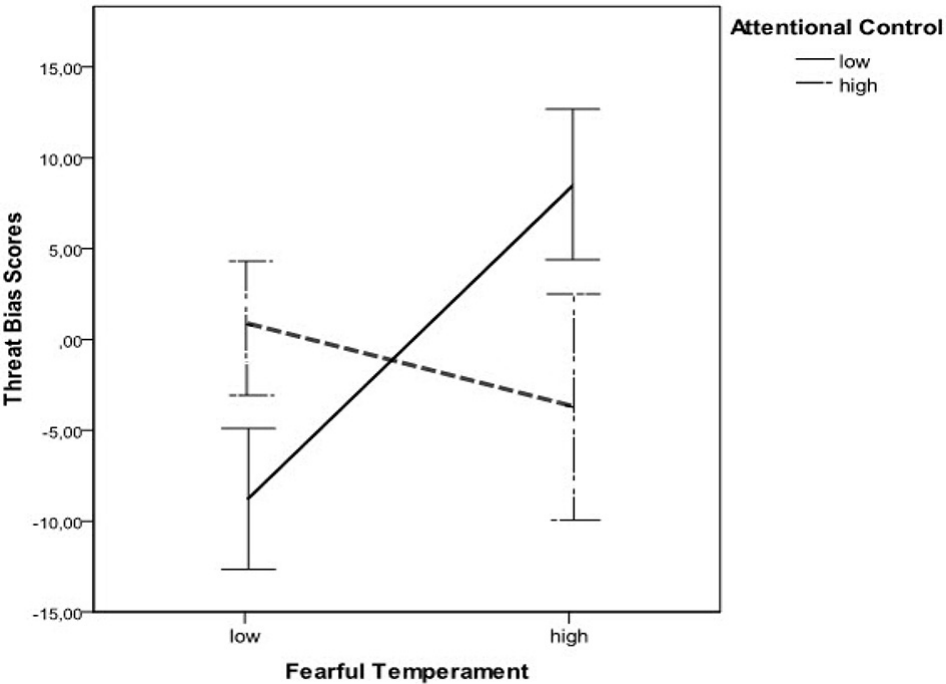
**Interaction effect of temperamental fear and temperamental attentional control on threat bias scores (bars represent values of standard errors)**.

We also ran several one-sample *t*-tests in order to compare bias scores for each group to 0. When bias scores are significantly different from 0, they indicate a clear attentional bias. For the low fear, low attentional control group, the mean bias score was significantly different from 0, *t*_(38)_ = −2.26, *p* = 0.02, *d* = 0.51. The same was true for the high fear, low attentional control group *t*_(42)_ = 2.06, *p* = 0.04, *d* = 0.45. In the low fear high attentional control group, the mean bias score was not significantly different from 0, *t*_(52)_ = 0.09, *ns*. Also, the mean bias score did not significantly differ from 0 in the high fear, high attentional control group, *t*_(22)_ = −0.58, *ns*. Consequently, attentional biases appear to be present in the two groups of children that have low attentional control, at both high and low levels of fear. Specifically, children with high fear and low attentional control are significantly vigilant toward angry faces, whereas children with low fear and low attentional control present a significant attentional avoidance of angry faces. Children high in attentional control, with either low or high levels of fear, are not significantly biased in their attentional responses when confronted with an angry face (see Figure 1).

We conducted a second ANCOVA for the happy-neutral trials. We looked for possible effects of Fear and Attentional Control on bias scores for the happy-neutral stimuli, also controlling for the effects of Age and Anxiety. Results indicated no main effect of Fear, *F*_(3, 152)_ = 0.004, *ns*., Attentional Control, *F*_(3, 152)_ = 2.99, *ns*., and no interaction effect, *F*_(3, 152)_ = 0.23, *ns*. Also, the effects of Age *F*_(3, 152)_ = 0.19, *ns*., and Anxiety *F*_(3, 152)_ = 0.24, *ns*., did not reach significance. Therefore, it seems that the relation between fear, attentional control, and attentional biases is not a significant one in the case of happy faces.

#### Regression analyses

Because both fear and attentional control were measured on a continuous scale, we conducted an additional analysis based on hierarchical regression, in order to test the interaction between these two variables in predicting attentional biases toward angry faces. In addition, another potential difficulty in using ANCOVA arises from the use of correlated fear and attentional control measures (*r* = −0.30 in this sample), which may lead to inflated ANCOVA interaction if dichotomous groups are formed through median splits (Derryberry and Reed, 2002).

Therefore, hierarchical regression has the advantage of overcoming the problems of dichotomization of continuous variables based on median split procedures (Cohen et al., 2003). Following Aiken and West's (1991) guidelines, all variables were first centered and the interaction term (Fear × Attentional control) was computed as the multiplicative product of these two centered variables. Age and Anxiety were first entered. Fear was entered in the second step, followed by the Attentional control in the third step. The interaction term was entered in the fourth step.

**Figure 2:**
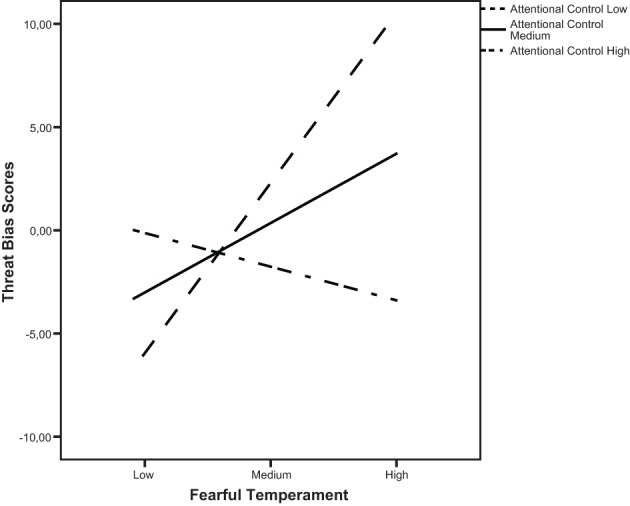
**The regression of threat bias scores on fearful temperament and attentional control (straight lines represent expected scores)**.

Consistent with the results from ANCOVA, this analysis (see Table 3) yielded a significant Fear x Attentional control interaction on step forth (*b* = −1.39, *p* = 0.01, *f*^2^ = 0.06). However, steps 1–4 were not significant (all *ps* > 0.05). We examined the particular form of this interaction by plotting the regression of threat bias scores on temperamental fear at high (one standard deviation above the mean), medium, and low (one standard deviation below the mean) levels of fear and attentional control. As shown in Figure 2, the slope was significantly different from zero only at low levels of attentional control, *t*_(154)_ = 2.73, *p* < 0.01. More specifically, there was a significant positive association between fear and attentional biases toward angry faces only for children with low attentional control. At high or medium values the slopes were not significantly different from zero, *t*_(154)_ = −55, *p* = 0.57 and *t*_(154)_ = 1.63, *p* = 0.10. These results indicate that there is no significant relation between temperamental fear and attentional vigilance toward threatening stimuli for children with good abilities for attentional control.

**Table 3:** **Summary of hierarchical regression analysis for variables predicting attentional biases toward angry faces**.

**Predictor**	**Δ *R*^2^**	***SE b***	***B***
Step 1	0.01		
Age (in months)		0.15	0.01
Anxiety		0.14	0.20
Step 2	0.02		
Fear		3.48	3.24
Step 3	0.02		
Attentional control		0.49	−0.39
Step 4	0.06		
Fear × attentional control		0.56	−1.39^*^
Total *R*^2^	0.11		

*N = 158*;

* *p < 0.05*.

Consistent with the results from ANCOVA, no significant results were found for fear (*b* = 2.81, *p* = 0.41), attentional control (*b* = −30, *p* = 0.52), or interaction term (Fear × Attentional control *b* = 0.70, *p* = 0.21) in explaining attentional biases toward happy faces.

***Differentiating engagement and difficulty to disengage in the Dot-probe task***. As Koster and colleagues have pointed out, by comparing neutral-neutral trials in the Dot-probe task separately to congruent, respectively incongruent emotional-neutral trials, one could separate two components of attentional biases taking the form of heightened vigilance toward threat: faster engagement vs. difficulty of disengagement (Koster et al., 2004). Therefore, we also computed engagement and disengagement bias scores and conducted two separate hierarchical regressions to pinpoint the attentional mechanism responsible for the tendency of high fear low attentional control children to manifest greater attentional biases to threat. Engagement bias score reflects a faster response on congruent angry trials compared to neutral trials. This faster response is considered to show that individuals were preferentially holding their attention at the location of the angry face. Disengagement bias score reflects higher reaction times on the incongruent angry trials, due to the time needed to shift attention from the angry to the neutral location. We employed the following formulas in order to calculate these bias scores:

Engagement score = (Neutral-neutral trials reaction time) − (Congruent trials reaction time)
Disengagement score = (Incongruent trials reaction time) − (Neutral-neutral trials reaction time)

We also conducted two regression analyses for the engagement score, respectively disengagement score.

The regression analysis for the engagement score yielded a significant Fear × Attentional control interaction on the fourth step (*b* = −1.37, *p* = 0.01, *f*^2^ = 0.06). Also, steps 1–4 were not significant (all *ps* > 0.05). In the regression analysis for the disengagement score no significant results were found for fear (*b* = −3.14, *ns*), attentional control (*b* = −0.15, *ns*), or interaction term (Fear × Attentional control *b* = 0.001, *ns*) in explaining difficulty to disengage from angry faces. Therefore, it seems that only the faster engagement to threat is implicated in the variations of attentional biases as a factor of temperamental fear and temperamental attentional control in the current study.

## Discussion

The present study aimed to investigate the effects of individual differences in temperamental fear and temperamental attentional control on attention allocation toward threat. Specifically, we analyzed the role of attentional control in regulating threat-related attentional biases in children with high levels of temperamental fear.

With regard to the main effects of both temperamental variables on attentional biases toward angry faces, neither fear nor attentional control was significantly related to attentional biases. However, consistent with our prediction, we found a significant interaction effect of fear and attentional control on attentional allocation toward threat. In particular, children with low levels of attentional control and high levels of fear displayed a stronger vigilance bias toward angry faces, compared to children who have low levels of attentional control and also low levels of fear. This vigilance seems to be underlained by an enhanced engagement of attention by angry faces, as it is proved by our additional regression analysis conducted on the two components of bias scores identified following Koster et al. (2004). This result is consistent with theoretical accounts on attentional biases toward threat, that generally link both the automatic/pre-attentional threat detection mechanism and the disruption of effortful strategies such as temperamental attentional control, with enhanced engagement of attention by angry faces (Beck and Clark, 1997; Mathews and MacKintosh, 1998; Mogg and Bradley, 1998; Cisler and Koster, 2010). But the lack of a significant difference between children with low fear, high attentional control and children with high fear, high attentional control indicates that when attentional control is increased, high levels of fear are not associated with biased attention toward angry faces. Also, the one-sample *t*-tests analysis comparing bias scores to 0 showed that children with high fear and low attentional control were indeed significantly vigilant toward angry faces. In addition, this analysis demonstrated that the group of children with low fear and low attentional control displayed a significant bias away from angry faces. Therefore, it seems that low attentional control is a key variable associated with biased attentional allocation in relation to angry facial expressions. We also noted that children high in both fear and attentional control did not show a significant bias.

The significant interaction between fear and attentional control replicates earlier findings, showing that attentional biases toward threat were present only in children who had both high levels of negative affectivity, such as fear, and low levels of regulative temperamental traits, such as attentional control (Helzer et al., 2009; Lonigan and Vasey, 2009). Our results revealed that, in highly fearful children the modulating role of high temperamental attentional control is reflected by a tendency to display attentional avoidance in the presence of threatening information. This attentional avoidance may involve a substantial voluntary component, relative to attentional vigilance toward threatening stimuli that accompanied the response of highly fearful children with low abilities of attentional control. Fearful children might be thought of as particularly vulnerable to automatically orient their attention toward threatening stimuli in the environment. But our results, in line with previous findings mentioned above, point out that in circumstances when attentional control can be employed to inhibit the orientation of attentional resources toward threat, only a subset of fearful children (those with low attentional control) go on to exhibit this reactive attentional response.

To our knowledge, this study is one of the first to investigate the possibility that individual differences in attentional control might modulate threat-related biases in fearful children, when ecological stimuli, such as emotional faces, are presented. A similar approach with a pictorial Dot-probe detection task, but with stimuli selected from the International Affective Picture System (Lang et al., 2005), is that of Vervoort et al. (2011). These authors examined the links between reactive temperament (negative affectivity as a composite factor), regulative temperament (effortful control as a composite factor), attentional biases and internalizing problems, in adolescents with and without anxiety disorders. Of direct relevance to our study, initial attentional biases (e.g., when stimulus duration was 500 ms) were predicted neither by the negative affectivity—effortful control interaction, nor by a main effects model, in either group. In addition, when stimulus duration was 1250 ms, higher levels of effortful control were related to attentional biases away from threat, but only in the non-anxious group, whereas in the anxious group effortful control had almost no influence on attentional biases. Our results complement these data by demonstrating that, in a non-clinical sample of children, the regulative temperamental trait, here more specifically assessed as attentional control influenced the threat-related attentional biases pattern, as children with both high levels of attentional control and also high levels of fear manifested a pattern of attentional avoidance in relation to threatening stimuli. The added value of the present results is reflected in the finding that attentional control influences initial attentional biases, at least toward angry faces, since a stimulus duration of 500 ms is assumed to reflect early initial attention (Bradley et al., 2000). We observed attentional bias scores significantly different from 0 in the two groups of children characterized by low levels of attentional control. Thus, our data support the conclusion that individual differences in attentional control have to be considered when investigating threat-related attentional biases in children with non-clinical anxiety. Not taking this variable into consideration might explain the divergent pattern of results obtained in previous studies (Lonigan and Vasey, 2009).

Another important aspect was the lack of any moderating effects of age or anxiety level. The lack of an age effect is similar to results of other studies with different age groups (e.g., Hadwin et al., 2009; Lonigan and Vasey, 2009). However, it is divergent from the results of a recent study on trait anxiety in children, showing a main effect of age on emotional processing and a moderating effect of age on attentional biases for negative stimuli, in a modified Stroop task (Reinholdt-Dunne et al., 2012). Interestingly, their study included a wider age range (7–14) than ours (9–14), therefore the lack of age-related effects in our data does not rule out the possibility of differential emotional or, more specifically, threat processing in younger children.

In the present study, anxiety symptoms did not influence attentional bias scores. This is somewhat similar to the lack of attentional biases for threat in anxious children, reported by Reinholdt-Dunne and collaborators in the case of older children (mean age 11) (Reinholdt-Dunne et al., 2012). One possibility is that the lack of association between anxiety and attentional biases was due to the non-clinical sample involved in the current study, a point also made by Reinholdt-Dunne and colleagues in reference to their results. This explanation is supported by the failure of some previous studies conducted with non-clinical samples to find evidence for an association between high levels of anxiety (e.g., high levels of trait anxiety) and biases toward threat (Eschenbeck et al., 2004; Helzer et al., 2009). Also, there are studies which suggest that moderate to severe levels of clinical anxiety in children are reliably associated with increased attentional biases toward angry faces relative to neutral faces (Waters et al., 2010a). An alternative explanation is that, for children with non-clinical anxiety, the emotional reactivity related to anticipation of stress, derived from temperament fear, might influence the direction of attention to threatening information more than anxiety. This finding requires replication by including the assessment of both reactive temperamental fear and anxiety symptoms in future studies that investigate attentional biases with non-clinical samples. Moreover, it should be mentioned that our study was designed to evaluate whether there would be group differences in attentional biases, as a function of temperamental traits and their interaction. Thus, we did not preselect our sample based on extreme anxiety scores. Therefore, the absence of a relation between anxiety and bias scores from the present study does not indicate that the full model proposed by Lonigan and colleagues regarding the relations between temperament, attentional biases and anxiety is not plausible. Future studies should analyze the stability and change over time of these relations in a longitudinal design. However, the present findings provide evidence only for a relation between temperament and attentional biases.

In our study we also examined attentional biases for happy faces. However, we did not formulate any specific predictions regarding the direction of attentional processes for happy faces, given that some studies conducted with children (Waters et al., 2008) have found a bias toward this kind of stimuli, whereas others have not (Telzer et al., 2008). The analysis of happy-neutral trials revealed no relation between attentional biases for happy faces and temperamental traits. This result is in line with previous studies that revealed no attentional biases in relation to happy facial expressions in anxious youths or in children with underlying anxiety predispositions (Roy et al., 2008; Telzer et al., 2008).

There are several limitations to be considered when interpreting our current findings. First, temperamental traits and attentional biases were assessed concurrently. Therefore, no conclusion can be inferred regarding the directionality of the observed effects. From a developmental perspective, it is important to shed light on the specific ways these variables influence each other, so that longitudinal studies assessing these factors will be needed. Second, our study investigated only one part of the model formulated by Lonigan and his collaborators. In order to adequately test the full model, data should be collected longitudinally. For example, future studies should analyze the impact of attentional biases on anxiety symptoms in children with certain temperamental characteristics. Third, as this study included only children without anxiety disorders, the observed effects cannot be generalized to clinically anxious children, for whom the nature of attentional processes and their relations with temperamental traits may be different (Vervoort et al., 2011). Moreover, given that we used self-report instruments for both temperamental fear and attentional control, it would be important to complement such measurements in future studies, for example with a behavioral task for attentional control. In the present study, we tried to overcome the problem of correlated fear and attentional control measures by also conducting a hierarchical regression in order to analyze our data. An additional aspect to be noted here is connected to the methodological weaknesses of the Dot-probe paradigm. It has been pointed out that reaction time effects in this task could be due to behavioral interference rather than to attentional phenomenon *per se*, especially at longer stimulus durations (e.g., Wolters et al., 2012). We tried to control for such confounds by running a preliminary analysis, to compare reaction times on all neutral faces trials to reaction times on all angry, respectively all happy faces trials, which showed no significant differences. However, it remains open to discussion whether the generally accepted calculation of bias scores in this task can accurately differentiate between attentional vigilance and avoidance (with positive bias scores indicating vigilance and negative ones indicating avoidance). This is because, during the 500 ms stimulus presentation interval, several shifts of attention are possible (Weierich et al., 2008). Thus, without systematic variation in display time and/or eye movements monitoring, it is virtually impossible to be certain what reaction times stand for, in terms of attentional vigilance vs. avoidance, at the end of the 500 ms interval. Therefore, conclusions regarding the presence of attentional biases of vigilance toward threat as opposed to avoidance of threat are to be regarded with caution. It is very important that future reaction times studies strive to provide better control in pinpointing the time course of attentional shifts.

In conclusion, despite the inherent limitations, the present results point to the importance of studying threat-related attentional biases in relation to temperamental traits. Results indicate that heightened vigilance toward angry faces is characteristic only of children with high fear and low attentional control.

The present study indicates that temperamentally-based attentional control plays a regulative role, modulating reactivity that characterizes temperamental fearfulness. Therefore, based on our data, we advance the hypothesis that attentional control can be seen as a possible early protective factor for the development of attentional biases toward threat, and further for the manifestation of anxiety problems. Future work, using a longitudinal design with both clinical and non-clinical samples, is required to examine this hypothesis.

### Conflict of interest statement

The authors declare that the research was conducted in the absence of any commercial or financial relationships that could be construed as a potential conflict of interest.

## Footnotes

^1^The MacArthur Foundation Research Network on Early Experience and Brain Development. (n.d.).

^2^We are grateful to Reviewer 1 for suggesting this additional data collection in order to validate the emotional face stimuli used in the Dot-Probe task.

^3^The ANCOVA analysis was also conducted with the addition of Gender as a between variable (resulting in a 2 × 2 × 2 ANCOVA). However, results indicated no significant main effect of Gender, *F*_(5, 150)_ = 0.40, *ns*, or interaction effects of Gender with Fear and Attentional control, *F*_(5, 150)_ = 0.70, *ns*.
