# Supplementary material for: Child temperamental reactivity and self-regulation effects on attentional biases
Source: Front Psychol. 2014 Aug 25;5:922. doi: 10.3389/fpsyg.2014.00922 (PMC4142332; doi:10.3389/fpsyg.2014.00922)
Supplement: Supplementary file 1 [file DataSheet1.PDF]

Supplementary Material

Child temperamental reactivity and self-regulation effects on attentional biases

Georgiana Susa[1], Oana Benga[1]*, Irina Pitica[1], Mircea Miclea[2]

[1]Developmental Psychology Lab, Psychology Department, Babes-Bolyai University, Cluj-Napoca, Romania
[2] Psychology Department, Babes-Bolyai University, Cluj-Napoca, Romania

*** Correspondence: Oana Benga,** Developmental Psychology Lab, Psychology Department, Babes-Bolyai University, Republicii, Cluj-Napoca, 400015, Romania. oanabenga@gmail.com

Supplementary Data

In order to additionally validate our results, we recruited a second group of children having, the same age as participants from our initial sample, and we tested whether they can accurately identify the emotional meaning (i.e., recognition accuracy) and rate the emotional intensity of the facial stimuli used within the Dot-Probe task.[1]

1. Method

1.1. Participants

Data were collected from 20 children (10 boys), age range 9-11 years (M= 133.90; SD=16.06), from three local schools that were collaborating with our Laboratory within this academic year. Due to the limited time frame (in Romania, school year ends on 20th of June, while this pilot study started in the beginning of June), we were not able to recruit a larger sample of children.

1.2. Stimuli

Stimuli were images from the NimStim, Ekman and Mogg & Bradley sets, producing a total N of 128 of photographs. This total N was obtained because each individual selected from a set posed 2 expressions: emotional (happy or angry) and neutral. Therefore, we had 22 different actors from NimStim, who each posed 2 expressions (22 individuals x 2 expressions); the same was true for both Ekman (5 individuals x 2 expressions) and Mogg & Bradley (37 individuals x 2 expressions) stimuli.

1.3. Evaluation Procedure

Children were tested individually. Images were presented on a grey scale in a printed high-quality format preserving the same size they had in the Dot-Probe task. For recognition accuracy, on each trial, a face was presented and participants were instructed to look carefully at the photograph and to

[1]We are grateful to Reviewer 1 for suggesting this additional data collection in order to validate the emotional face stimuli used in the Dot-Probe task.

say which emotion among the five choices (angry, happy, fear, neutral, none of the above) was expressed on the face. For emotional intensity, participants were asked to rate on a scale from 0 (not at all) to 6 (extremely intense) how intense they perceive the emotion expressed on the face. Stimuli were presented randomly, in a different order for each participant, and participants proceeded at their own pace (this procedure was adapted from Tottenham et al., 2009).The researcher recorded the participant's responses on a standardized response sheet, writing the code of the photograph, the emotion chosen by the participant and the intensity rating.

2. Results

In Table S1 presented below, we report % of agreement for valence, as well as means and standard deviations for intensity ratings, for the whole 128 stimuli used in the Dot Probe task. We would like to mention that higher S.D. for neutral faces intensity ratings are due to the fact that only a few participants rated each neutral face with a value greater than 0.

Table S1

Percentage Agreement for Valence, Means and Standard Deviations for Intensity Ratings Corresponding to Each Facial Stimulus from the Three Sets

**Neutral**			**Angry**			**Happy**		
Picture ID	Valence	Intensity Mean (*SD*)	Picture ID	Valence	Intensity Mean (*SD*)	Picture ID	Valence	Intensity Mean (*SD*)
**1Fe_Mogg**	80%	0.35 (.67)	**1Fe_Nim**	100%	3.33 (1.45)	**1Ma_Mogg**	100%	4.05 (1.23)
**2Fe_Mogg**	90%	0.30 (.97)	**2Ma_Mogg**	100%	5.50 (0.51)	**2Ma_Mogg**	100%	3.85 (1.22)
**3Fe_Nim**	75%	0.55 (.88)	**3Fe_Mogg**	90%	3.80 (1.23)	**3Ma_Mogg**	100%	4.80 (1.10)
**4Fe_Nim**	85%	0.40 (.88)	**4Ma_Mogg**	100%	4.95 (1.09)	**4Fe_Nim**	100%	4.55 (1.09)
**5Fe_Nim**	90%	0.45 (1.14)	**5Fe_Mogg**	100%	5.60 (1.14)	**5Fe_Ekman**	100%	3.75 (0.96)
**6Ma_Mogg**	90%	0.20 (0.69)	**6Fe_Mogg**	90%	3.10 (1.25)	**6Ma_Mogg**	100%	3.75 (1.06)
**7Fe_Nim**	100%	0.20 (0.89)	**7Ma_Mogg**	100%	3.70 (1.41)	**7Fe_Mogg**	100%	4.70 (0.86)
**8Ma_Mogg**	100%	0 (0)	**8Fe_Nim**	100%	5.80 (0.41)	**8Fe_Mogg**	100%	3.45 (1.39)
**9Ma_Mogg**	100%	0.25 (0.63)	**9Ma_Nim**	90%	3.25 (1.01)	**9Fe_Mogg**	90%	5.15 (0.74)
**10Fe_Nim**	90%	0.20 (0.69)	**10Ma_Mogg**	90%	4.20 (1.36)	**10Fe_Mogg**	100%	5 (0.72)
**11Ma_Mogg**	90%	0.45 (1.09)	**11Ma_Mogg**	100%	4.20 (1.15)	**11Ma_Nim**	100%	4.40 (1.14)
**12Fe_Nim**	90%	0.45 (1.05)	**12Fe_Mogg**	80%	3.25 (0.85)	**12Ma_Ekman**	100%	5.20 (1.76)
**13Ma_Nim**	100%	0 (0)	**13Ma_Mogg**	100%	4.80 (1.23)	**13Fe_Mogg**	100%	3.25 (1.01)
**14Ma_Mogg**	90%	0.10 (0.30)	**14Ma_Mogg**	100%	4.60 (1.14)	**14Fe_Mogg**	100%	5.10 (0.85)
**15Fe_Nim**	100%	0 (0)	**15Fe_Mogg**	95%	3.30 (1.30)	**15Fe_Nim**	100%	4.10 (1.20)
**16Ma_Mogg**	100%	0 (0)	**16Fe_Nim**	100%	4.90 (1.29)	**16Fe_Nim**	100%	5.35 (0.81)
**17Ma_Mogg**	90%	0.35 (0.81)	**17Ma_Nim**	100%	5.05 (1.14)	**17Ma_Nim**	100%	3.75 (0.91)
**18Ma_Mogg**	90%	0.35 (0.87)	**18Fe_Mogg**	90%	3.40 (1.56)	**18Ma_Mogg**	100%	4.65 (0.81)
**19Ma_Mogg**	100%	0 (0)	**19Ma_Nim**	100%	5.10 (1.25)	**19Ma_Mogg**	100%	5.30 (0.47)
**20Fe_Nim**	100%	0 (0)	**20Ma_Nim**	100%	5.45 (0.99)	**20Fe_Nim**	100%	4.95 (0.82)
**21Ma_Nim**	100%	0 (0)	**21Fe_Ekman**	90%	3.95 (1.19)	**21Ma_Mogg**	100%	4.05 (0.82)
**22Fe_Ekman**	100%	0 (0)	**22Fe_Nim**	100%	4.05 (1.53)	**22Ma_Nim**	100%	3.70 (0.97)
**23Fe_Mogg**	100%	0 (0)	**23Ma_Nim**	90%	4.60 (1.39)	**23Ma_Ekman**	100%	5.35 (0.93)
**24Ma_Nim**	100%	0 (0)	**24Ma_Mogg**	100%	4.15 (1.30)	**24Fe_Mogg**	95%	5.20 (0.69)
**25Ma_Nim**	100%	0 (0)	**25Ma_Nim**	100%	5.20 (1.19)	**25Fe_Mogg**	100%	3.85 (1.38)

**26Ma_Mogg**	100%	0 (0)	**26Ma_Nim**	100%	5.10 (1.37)	**26Fe_Mogg**	100%	4.60 (1.09)
**27Fe_Mogg**	80%	0.50 (0.88)	**27Ma_Nim**	100%	5.15 (1.78)	**27Ma_Mogg**	100%	4.25 (1.51)
**28Fe_Mogg**	80%	0.30 (0.65)	**28Fe_Nim**	100%	4.40 (1.60)	**28Ma_Mogg**	100%	4.05 (1.19)
**29Ma_Mogg**	90%	0.16 (0.45)	**29Fe_Mogg**	90%	5 (1.21)	**29 Ma_Mogg**	100%	3.15 (0.98)
**30Fe_Mogg**	90%	0.15 (0.48)	**30Fe_Ekman**	100%	3.65 (1.38)	**30Ma_Nim**	100%	3.50 (1.05)
**31Fe_Mogg**	80%	0.25 (0.55)	**31Fe_Mogg**	100%	4.70 (1.75)	**31Fe_Mogg**	100%	5.25 (0.55)
**32Fe_Mogg**	90%	0.15 (0.49)	**32Fe_Mogg**	100%	4.05 (1.57)	**32Fe_Nim**	100%	5.25 (0.71)
**33Fe_Mogg**	100%	0 (0)						
**34Fe_Mogg**	90%	0.10 (0.30)						
**35Fe_Mogg**	85%	0.40 (0.88)						
**36Ma_Mogg**	80%	0.25 (0.55)						
**37Fe_Mogg**	90%	0.15 (0.48)						
**38Ma_Mogg**	100%	0 (0)						
**39Fe_Mogg**	100%	0 (0)						
**40Fe_Mogg**	100%	0 (0)						
**41Fe_Mogg**	75%	0.60 (0.94)						
**42Ma_Mogg**	90%	0.10 (0.30)						
**43Fe_Mogg**	100%	0 (0)						
**44Ma_Mogg**	80%	0.30 (0.57)						
**45Ma_Mogg**	80%	0.30 (0.65)						
**46Ma_Nim**	100%	0.15 (0.67)						
**47Ma_Mogg**	100%	0 (0)						
**48Ma_Nim**	100%	0 (0)						
**49Ma_Nim**	100%	0 (0)						
**50Ma_Nim**	90%	0.25 (0.78)						
**51Ma_Nim**	90%	0.15 (0.48)						
**52Fe_Ekman**	80%	0.40 (0.88)						
**53Fe_Mogg**	75%	0.30 (0.57)						
**54Ma_Nim**	100%	0 (0)						
**55Fe_Nim**	90%	0.15 (0.36)						
**56Fe_Nim**	90%	0.40 (0.75)						
**57Fe_Ekman**	100%	0 (0)						
**58Fe_Mogg**	85%	0.20 (0.52)						
**59Ma_Nim**	90%	0.15 (0.48)						
**60Ma_Ekman**	100%	0 (0)						
**61Ma_Ekman**	100%	0.15 (0.36)						
**62Ma_Mogg**	100%	0.15 (0.48)						
**63Fe_Mogg**	80%	0.30 (0.57)						
**64Ma_Nim**	100%	0 (0)						

References

Tottenham, N., Tanaka, J., Leon, A. C., McCarry, T., Nurse, M., Hare, T. A.,… Nelson, (2009). The NimStim set of facial expressions: judgements from untrained research participants. *Psychiatry Research*, *168*(3), 242-249. doi:10.1016/j.psychres.2008.05.006
